# Impairments in the Initiation of Maternal Behavior in Oxytocin Receptor Knockout Mice

**DOI:** 10.1371/journal.pone.0098839

**Published:** 2014-06-03

**Authors:** Megan E. Rich, Emily J. deCárdenas, Heon-Jin Lee, Heather K. Caldwell

**Affiliations:** 1 Laboratory of Neuroendocrinology and Behavior, Department of Biological Sciences, Kent State University, Kent, Ohio, United States of America; 2 School of Biomedical Sciences, Kent State University, Kent, Ohio, United States of America; 3 Department of Oral Microbiology, School of Dentistry and the Brain Science and Engineering Institute, Kyungpook National University, Daegu, South Korea; Max Planck Institute of Psychiatry, Germany

## Abstract

Oxytocin (Oxt) acting through its single receptor subtype, the Oxtr, is important for the coordination of physiology and behavior associated with parturition and maternal care. Knockout mouse models have been helpful in exploring the contributions of Oxt to maternal behavior, including total body *Oxt* knockout (Oxt −/−) mice, forebrain conditional *Oxtr* knockout (Oxtr FB/FB) mice, and total body *Oxtr* knockout (Oxtr −/−) mice. Since Oxtr −/− mice are unable to lactate, maternal behavior has only been examined in virgin females, or in dams within a few hours of parturition, and there have been no studies that have examined their anxiety-like and depression-like behavior following parturition. To improve our understanding of how the absence of Oxt signaling affects maternal behavior, mood and anxiety, we designed a study using Oxtr −/− mice that separated nursing behavior from other aspects of maternal care, such as licking and grooming by thelectomizing (i.e. removing the nipples) of Oxtr +/+ mice and sham-thelectomizing Oxtr −/− mice, and pairing both genotypes with a wet nurse. We then measured pup abandonment, maternal behavior, and postpartum anxiety-like and depression-like behaviors. We *hypothesized* that genetic disruption of the *Oxtr* would impact maternal care, mood and anxiety. Specifically, we predicted that Oxtr −/− dams would have impaired maternal care and increased anxiety-like and depression-like behaviors in the postpartum period. We found that Oxtr −/− dams had significantly higher levels of pup abandonment compared to controls, which is consistent with previous work in Oxtr FB/FB mice. Interestingly, Oxtr −/− dams that initiated maternal care did not differ from wildtype controls in measures of maternal behavior. We also did not find any evidence of altered anxiety-like or depressive-like behavior in the postpartum period of Oxtr −/− dams. Thus, our data suggest that Oxt lowers the threshold for the initiation of maternal behavior.

## Introduction

In females, the neuropeptide oxytocin (Oxt) helps to coordinate uterine contractions and milk ejection (for review see[1], [2]). Given these peripheral effects, it is not surprising that central Oxt coordinates aspects of maternal behavior (for review see [3]). During the peripartum period there are increases in Oxt release in the periphery [4]–[6], increased expression in the paraventricular (PVN) and supraoptic (SON) nuclei, [7]–[9], and increased release in the hypothalamic and limbic regions of the brain [10]–[13]. There are also concurrent increases in Oxt receptor (Oxtr) expression in areas of the brain such as the PVN, SON, medial preoptic area (MPOA), bed nucleus of the stria terminalis (BNST), and lateral septum (LS); all of which are known to be important for the onset of maternal care [14]–[19]. Specifically, rat dams with higher levels of Oxtr expression in the BNST, MPOA, and LS display increased levels of pup licking, grooming, and arched-back nursing behaviors (High LG-ABN)[20], [21].

In addition to its importance to maternal behavior, Oxt in the postpartum period may impact anxiety and depression. One of the changes thought to be necessary for normal maternal behavior is decreased anxiety during the postpartum period [22]–[26], which allows for a mother to more easily accept offspring; in turn facilitating social bonding (for review see [27]). In both lactating and virgin rats, chronic intracerebroventricular (icv) infusion of Oxt reduces anxiety-like behavior, as measured by an increase in the amount of time spent in the open arms of an elevated plus maze relative to controls [28], [29]. Conversely, in rodents, icv infusion of selective Oxtr antagonists increases anxiety-like behavior and decreases maternal care [28], [30]. In humans, elevated anxiety during the postpartum period is associated with postpartum depression [31]–[37] and lower plasma Oxt concentrations during pregnancy are correlated with increased risk of postpartum depression [38], [39].

While elegant pharmacological work has provided insight into the neural regulation of maternal behavior and postpartum anxiety-like and depression-like behaviors [28], [29], [40]–[43], the use of genetic tools, including *Oxt* and *Oxtr* knockout mice (Oxt −/− and Oxtr −/−, respectively) have also made significant contributions. Initial studies in Oxt −/− mice revealed surprisingly normal parturition and maternal behavior, despite the inability to milk eject [44], [45]. However, more recent work in virgin Oxt −/− mice has found decreases in pup retrievals and pup licking [46]. Due in part to the discrepancies between observed findings in Oxt −/− dams, as well as evidence that arginine vasopressin (Avp) is able to signal through the Oxtr [47], Oxtr −/− mice were genetically engineered by two different groups to provide a more complete disruption of Oxt signaling [48], [49].

Unfortunately, there have been conflicting reports regarding the effects of Oxtr disruption on maternal behavior in these two lines of Oxtr −/− mice. Like Oxt −/− mice, Oxtr −/− females appear to have normal parturition and an inability to milk eject. In the Nishimori line of Oxtr −/− mice, virgin and postpartum females display longer latencies to retrieve pups and postpartum females display less time crouching over pups [49]; though maternal behaviors were only assessed on postnatal day 0 (PND0). These findings contrast with our line of Oxtr −/− mice, in which virgins appear to have normal maternal behavior [50]. Whether the aforementioned discrepancies between the two lines are due to differences in the background of the mice, or differences in behavioral testing, remains unknown. A study in forebrain conditional *Oxtr* knockout mice (Oxtr FB/FB) suggests that they have normal maternal behavior, but exhibit increases in pup mortality compared to Oxtr wildtype (+/+) mice [50]; suggesting that Oxtr activation specifically in the forebrain is important for the initiation of maternal care.

Previous research has also found that Oxtr −/− mice have deficits in social recognition [48], [49] and increased intermale aggression [49], [51]. However, anxiety-like behavior is reportedly normal compared to Oxtr +/+ mice [48]. To date there have been no published studies on anxiety-like and depression-like behaviors in postpartum female Oxtr −/− mice. Thus, to test the hypothesis that a lack of Oxt signaling throughout the entire body would affect maternal, anxiety-like, and depression-like behaviors in our line of Oxtr −/− mice, we designed experiments that separated nursing behavior from other aspects of maternal care. We thelectomized (i.e. removed the nipples) Oxtr +/+ mice, and sham-thelectomized Oxtr −/− mice, as Oxtr −/− mice are unable to milk eject, and paired each with a wet nurse. We were then able to measure maternal behavior and anxiety-like and depression-like behaviors in the absence of nursing. We predicted that a subset of Oxtr −/− females would abandon their pups, but that those who did not would have normal maternal behavior. We also predicted that Oxtr −/− dams would have increased anxiety-like and depression-like behaviors in the immediate postpartum period.

## Methods

### Animals and housing

Female siblings (19–20 days old) from heterozygous breeding pairs were weaned at 21 days and group housed until at least 8 weeks of age. Animals were maintained on a 12∶12 light-dark cycle (lights on 0200h) and food and water were available *ad libitum*. The Kent State University Animal Care and Use Committee approved all procedures. Genotyping of Oxtr +/+ and Oxtr −/− mice was performed as described previously [48].

### Thelectomy Surgery

Virgin Oxtr +/+ mice were thelectomized to eliminate milk ejection, and Oxtr −/− mice received a sham surgery, as they already cannot milk eject. Our choice to have Oxtr −/− mice only receive a sham surgery was guided by previous research indicating that suckled and non-suckled rat dams display similar levels of maternal behaviors and have similar c-fos expression in brain regions associated with the regulation of maternal behavior [52], [53]. Briefly, females were anesthetized using isoflurane, then an iodine solution, followed by 100% ethanol, were then applied to the nipple and surrounding tissue. For Oxtr +/+ mice, the nipple was retracted and removed at the base with sterilized scissors. Oxtr −/− mice were sham-thelectomized by making a small scissor cut lateral to each of the nipples. There was little to no bleeding after surgery; thus, sutures were not necessary. Antibacterial ointment was then applied to the areas and re-applied as needed once daily for 5 days post surgery. Following surgery, animals were assigned an identification that did not provide any genotype information; this ensured that the experimenter was blind to the genotypes of the animals for the remainder of testing.

At least 1 week post surgery, Oxtr +/+ (n = 19) and Oxtr −/− mice (n = 26) were group housed with wet nurses, as neither group of experimental mice were able to milk eject. Wet nurses were female C57BL/6J or females from our Flox/Flox line of mice, which are a mixture of C57BL/6J and 129/Sv. Wet nurse dams and experimental mice were paired for at least 1-week post surgery. 3 days prior to mating, male bedding was added to the females' cages to induce the Whitten effect, where male odors induce estrus and synchronize estrus cycles among females [54], [55]. Experimental Oxtr +/+ mice, Oxtr −/− mice, and wet nurse dams were grouped with a male for up to 7 days. All females were checked for sperm plugs for 5 days after the addition of the male to determine if mating had occurred and to estimate the timing of parturition. If a sperm plug was found, the male was removed. Females in which no sperm plugs were identified were paired again with a male until pregnancy resulted.

Approximately 1 week prior to the expected parturition date, individual experimental Oxtr +/+ and Oxtr −/− mice and their wet nurses were rearranged so that experimental animals and the wet nurses had the same projected parturition date. If necessary, Oxtr +/+ and Oxtr −/− mice were paired with an alternative wet nurse prior to parturition to ensure that the wet nurse experienced parturition on the same day or the day prior to Oxtr +/+ or Oxtr −/− mice. If an experimental dam gave birth prior to its paired wet nurse it was removed from the study, as the pups were unable to survive due to the lack of milk ejection in the experimental animals. On PND0, defined as the day of parturition, all wet nurse and experimental litters were collectively culled to a total of 4 pups. No attempt was made to discriminate between offspring from experimental versus wet nurse dams. Prior to testing on PND1, dams were checked for pups. Those dams that did have pups remaining were then tested for maternal care. If no pups remained it was noted whether pups were abandoned or cannibalized. Prior to all behavioral testing, animals were acclimated to the testing space for 1 hour, and unless otherwise indicated, tests were performed 1 hour after lights out under dim red light illumination.

### Experiment 1: Maternal Care and Aggressive Behavior

Oxtr +/+ (n = 9) and Oxtr −/− (n = 7) females were tested for maternal care on PND1-3. The wet nurse was removed for all portions of the maternal care testing, and the maximum amount of time the wet nurse was separated from the pups was approximately 20 minutes. Pup retrievals and maternal behaviors were recorded by first removing all pups from the Oxtr +/+ or Oxtr −/− dams for 5 minutes. Removed pups were placed into a cage with their wet nurse. After 5 minutes, the pups were scattered in the home cage opposite of the dam, and the dam's behavior was then videotaped for an additional 20 minutes and later quantified for maternal behaviors.

Pup retrievals were quantified in the first 5 minutes following the return of the pups to the cage, and included the latency to retrieve the first pup and the latency to retrieve all of the pups. Latency to retrieve all pups was determined by the amount of time the dam took to return all 4 pups to the nest. If not all pups were retrieved within 5 minutes of being returned to the home cage, a latency score of 300 s was recorded and maternal behaviors were not scored. If all the pups were retrieved, 10 minutes of maternal care was scored. Behaviors scored were: time on/off the nest, nest building, sniffing pups, licking pups, nursing position, self-grooming, digging, and non-social behaviors (eating, sleeping, and exploratory behaviors). All behaviors were coded using Noldus Observer 9.0 (Leesburg, VA) by an observer blind to the genotypes. Following testing, the wet nurse was returned to the home cage. Across the 3 days of testing there was a maximum of 15 minutes of searching behavior and 30 minutes of maternal care recorded for each animal. The duration of time the Oxtr +/+ and Oxtr −/− dams spent engaging in all the behaviors measured was summed, and maternal care and pup retrieval were analyzed within each day using a one-way ANOVA with genotype as the main factor. A repeated measures analysis was not performed because only some animals retrieved pups on PND1-3. Thus, we did not score maternal behaviors for all 3 days for all of the animals. A Fisher's exact test was used to compare pup mortality between Oxtr +/+ and Oxtr −/− mice. In all statistical tests a p-value of ≤0.05 was considered statistically significant.

Maternal aggression was tested in Oxtr +/+ and Oxtr −/− dams each day from PND4-6 1 hour after lights out under dim red light. Testing was completed on PND4-6 so it would not interfere with the maternal behavior testing, as we did not want to inadvertently cause an increase in pup abandonment. Wet nurse dams were removed for the duration of testing, and pups remained in their home cage. An intruder Balb/c (adult, gonad-intact) (The Jackson Laboratory, Bar Harbor, ME) or C57BL/6J (adult, gonad-intact) male mouse between the age of 2 and 6 months was added to the home cage and the animals were recorded. If no attack against the intruder occurred during the 5-minute test, a latency of 300 s was recorded. When an attack occurred, the dam's behavior was scored for an additional 2 minutes after the time of the initial attack. If an attack occurred, the behaviors scored were: tail rattle, fleeing, attack behavior (lunge-bite), aggression (pushing, chasing, or contact with the intruder), self-grooming, and non-social behavior (eating, sleeping, and exploratory behaviors). All behaviors were scored using Noldus Observer 9.0 (Leesburg, VA) by an observer blind to the genotypes. No statistical analyses were performed for maternal aggression, as too few experimental animals displayed aggressive behavior.

### Experiment 2: Postpartum Anxiety-like and Depression-like Behavior

#### Anxiety-like Behavior

Measurements of postpartum anxiety-like and depression-like behaviors were conducted on a separate group of Oxtr +/+ (n = 8) and Oxtr −/− (n = 9) dams on PND4-6. These animals underwent surgery as described above. PND4-6 were selected for testing because this was after maternal behavior had been established and we were less likely to facilitate pup abandonment. All Oxtr +/+ and Oxtr −/− dams were paired with a wet nurse for the duration of testing, and were tested regardless of whether or not they had abandoned their pups. An elevated plus maze test was performed on PND4 under ∼100 lux lighting using the mouse maze as previously described [56], [57] (San Diego Instruments, San Diego, CA). Mice were placed in the center of the plus maze facing the closed arms, and tracked using Noldus Ethovision (7.0) (Leesburg, VA) for 10 minutes. For analysis, the elevated plus maze was divided into 3 segments: open arms, closed arms, and the center section. To assess anxiety-like behavior, the amount of time spent in the open and closed arms was compared between genotypes. The duration of time spent in the open and closed arms was summed, and the percentage of time spent in each was determined. Anxiety-like behaviors were measured using a one-way ANOVA comparing time spent in open and closed arms and number of entries into the open arms, with genotype as the main factor. A p-value of ≤0.05 was considered statistically significant.

On PND5, experimental mice were tested in an open field. The open field arena was made out of Plexiglas and measured 45.5×45.5×30 cm [58]. Animals were placed into the center of the open field, and the animals were tracked for a total of 20 minutes under ∼200 lux lighting. Animals were tracked using Noldus Ethovision (7.0) (Leesburg, VA). The field was separated into 2 parts, an inner arena (measuring 32×32 cm), and outer arena. The animals were tracked to determine the amount of time spent in the inner versus outer arena. From this, the percentage of time spent in the inner and outer arenas was calculated. A one-way ANOVA was used to compare the percentage of time spent in the inner and outer arenas, as well as the number of entries into the inner arena, between genotypes. A p-value of ≤0.05 was considered statistically significant. Due to experimenter error, 1 Oxtr +/+ and 1 Oxtr −/− dam were removed from statistical analysis.

#### Depression-like Behavior

On PND6 Oxtr +/+ and Oxtr −/− mice were recorded in a forced swim test. They were placed into a 19 cm in diameter cylindrical tank filled to 24 cm with room temperature (∼21°C) water for 10 minutes, and observed for any signs of distress. Following testing, dams were returned to a clean cage with their wet nurse and pups. Forced swim was later scored using the Noldus Observer 9.0 (Leesburg, VA) as previously described [57], [58]. Briefly, swimming or floating was scored for 4 total minutes, with the scoring beginning at minute 2, to allow for the mouse to acclimate. A sampling method of scoring was conducted whereby every 5 seconds the behavior was scored as either a swim or a float. We have previously used this sampling method, as it reduces the observer error associated with the transitions [57], [58]. Swimming behavior was scored as 2 or more paws moving to propel the mouse. Float behavior was scored as no paws moving, or 2 or fewer paws moving slightly only to stabilize the mouse in the water. The number of float and swim behaviors scored were summed, and the percentage of swim and float behaviors scored was determined. A one-way ANOVA was used to compare the percent swim/float between genotypes. A p-value of ≤0.05 was considered statistically significant.

## Results

### Experiment 1: Maternal Care and Aggressive Behavior

**Figure 1:**
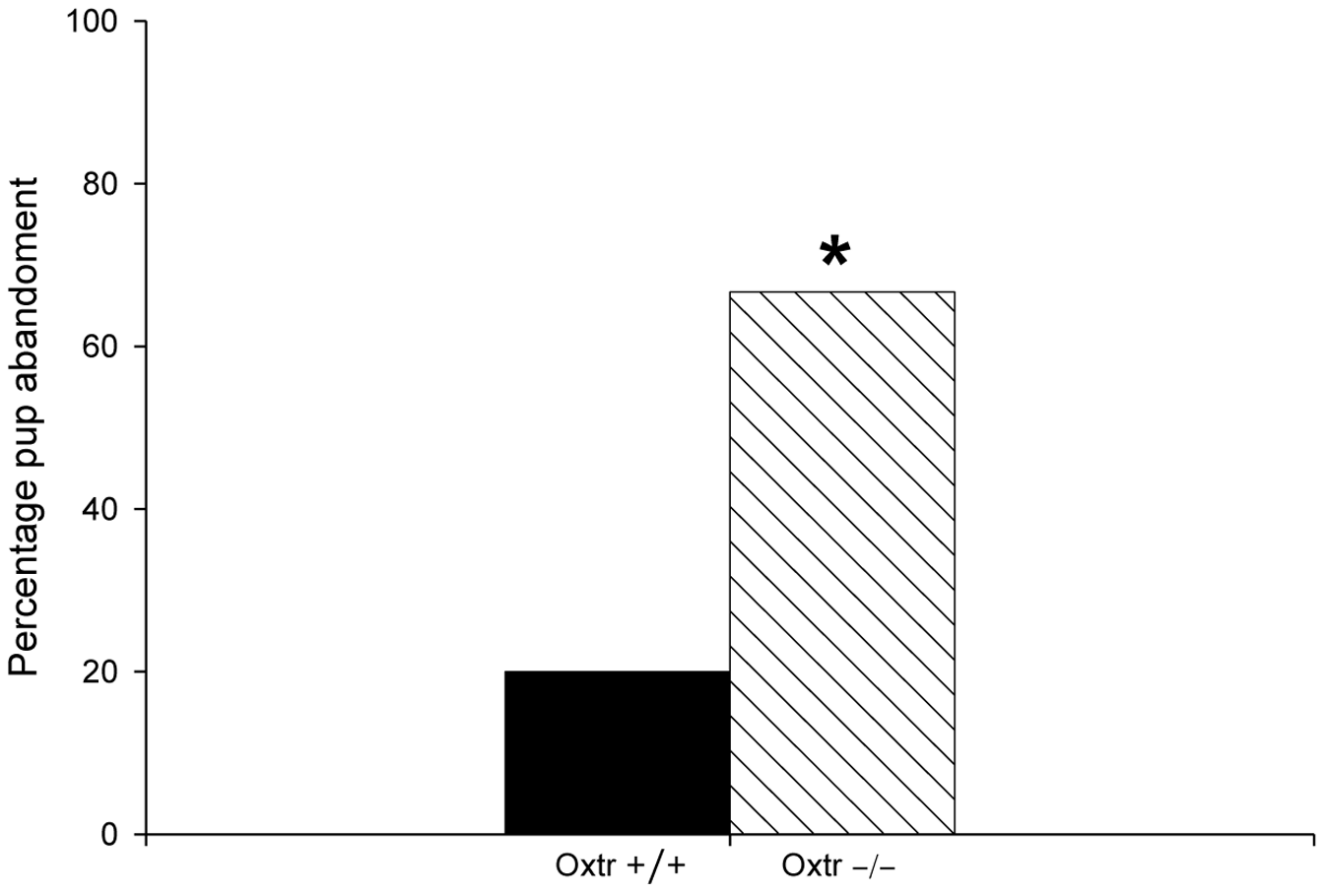
Increased pup abandonment was observed in Oxtr −/− dams compared to Oxtr +/+ dams. 67% of Oxtr −/− females cannibalized or abandoned their pups by PND1 compared to 20% of Oxtr +/+ females. *two-tailed p = 0.021.

**Figure 2:**
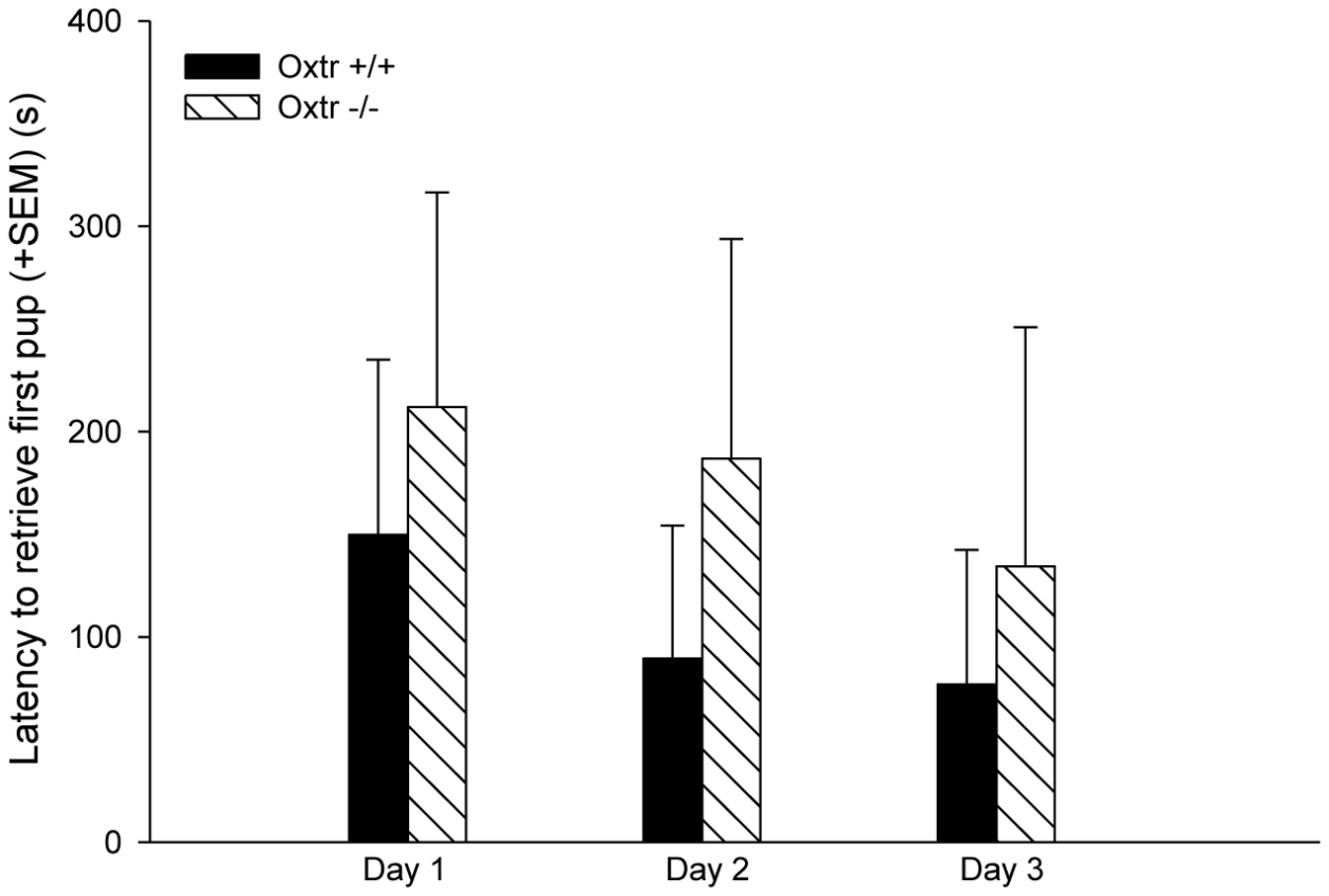
Latency to retrieve the first pup by Oxtr +/+ and Oxtr −/− dams on PND1-3. There were no genotypic differences in the latency to retrieve the first pup across the 3 days of testing.

10 out of 15 Oxtr −/− dams had abandoned their litters by PND1, with all pups cannibalized or found dead in the cages (See Figure 1). In contrast, only 2 of the 11 Oxtr +/+ females had abandoned their litters by PDN1 (See Figure 1)(two-tailed p = 0.021). Therefore, behavior from only 5 Oxtr −/− and 9 Oxtr +/+ dams was included in the maternal care and aggressive behavior experiment. There were no significant differences between genotypes in any of the maternal behaviors measured on PND1-3 (See Table 1). There were also no genotypic differences in the latency to retrieve the first pup and percentage of dams retrieving pups across days (See Figure 2). Furthermore, there was no difference in the time spent on the nest between Oxtr +/+ and Oxtr −/− females (See Table 1). On PND4 of the maternal aggression test only 3 out of 9 females fought (2 Oxtr +/+ females and 1 Oxtr −/− female), and on PND5-6 only 4 out of 9 females (3 Oxtr +/+ females and 1 Oxtr −/− female) displayed aggression, suggesting low levels of maternal aggression regardless of genotype. Since the numbers were low, no statistical analysis was completed.

**Table 1:** Mean Durations of Maternal Behavior (± SEM) on PND 1–3.

Day 1		
Behavior	Oxtr +/+ (mean ± SEM) (n = 9)	Oxtr −/− (mean ± SEM) (n = 7)
Time on nest	200.36±47.59	260.51±18.41
Time off nest	396.93±46.63	338.72±18.15
Duration Nest building	68.11±35.45	67.21±23.54
Duration Sniffing pup	12.85±5.44	9.93±8.60
Duration Licking pups	3.14±1.48	5.52±3.48
Duration Retrieving pups	11.13±9.35	1.69±1.67
Duration Self-grooming	11.13±3.67	16.93±3.86
Duration Non-social	490.61±35.92	497.38±30.41

Day 2		
Behavior	Oxtr +/+ (mean ± SEM) (n = 9)	Oxtr −/− (mean ± SEM) (n = 7)
Time on nest	257.80±58.58	243.34±48.13
Time off nest	341.69±58.59	356.09±48.08
Duration Nest building	82.04±33.40	89.46±48.96
Duration Sniffing pup	11.21±2.42	8.068±3.22
Duration Licking pups	6.92±2.03	20.60±9.60
Duration Retrieving pups	15.83±9.19	5.10±2.77
Duration Self-grooming	24.90±8.20	30.14±8.10
Duration Non-social	454.28±33.00	445.29±47.90

Day 3		
Behavior	Oxtr +/+ (mean ± SEM) (n = 9)	Oxtr −/− (mean ± SEM) (n = 7)
Time on nest	225.20±54.99	191.94±39.27
Time off nest	374.23±54.95	407.48±38.23
Duration Nest building	106.81±35.50	41.62±13.68
Duration Sniffing pup	8.23±3.09	5.46±2.92
Duration Licking pups	6.95±6.42	13.19±8.28
Duration Retrieving pups	9.57±9.57	9.13±8.40
Duration Self-grooming	10.67±4.32	3.2±0.80
Duration Non-social	469.48±35.30	498.94±25.26

### Experiment 2: Postpartum Anxiety-like and Depression-like Behavior

**Figure 3:**
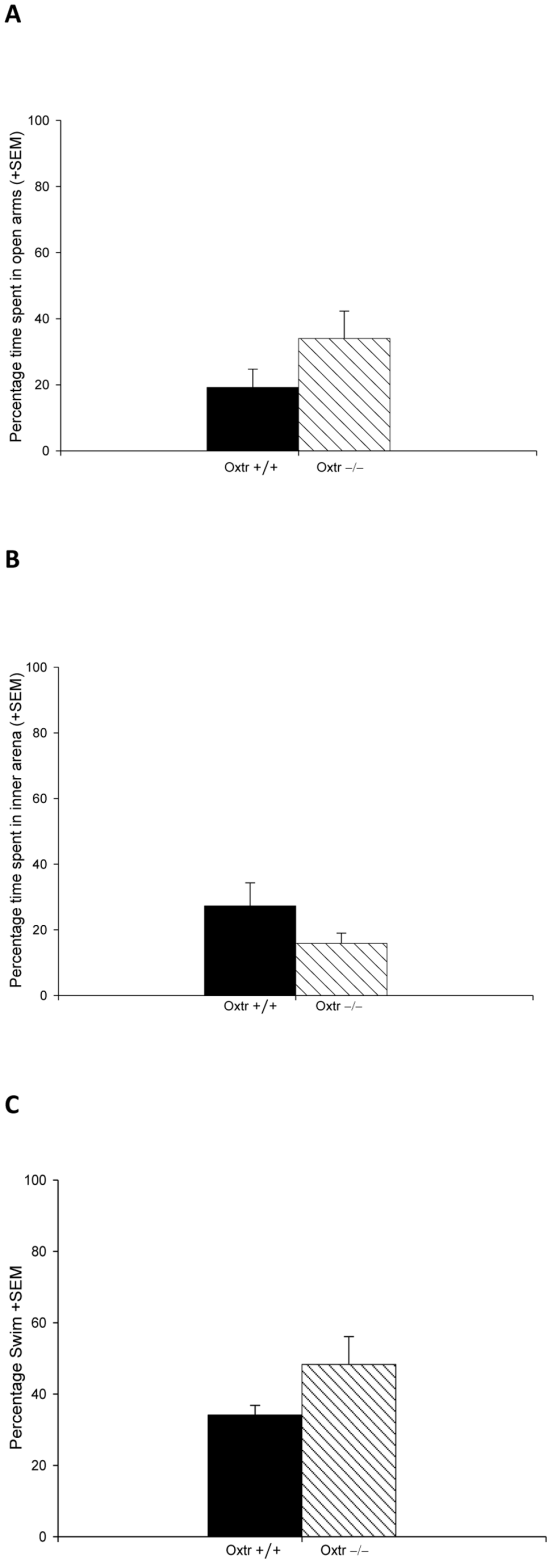
Oxtr −/− dams had normal anxiety-like and depression-like behavior, as measured by (A) percent time in the open arms compared to Oxtr +/+ dams in an elevated plus maze on PND4, (B) in percent time in the inner arena in an open field test on PND5 (B), and (C) in the percent time swimming in a forced swim test.

Oxtr −/− dams did not display increased anxiety-like and depression-like behaviors during the postpartum period compared to Oxtr +/+ dams. In the elevated plus maze, there was no genotypic difference between the percent time spent in the open and closed arms, F = _1,15_ = 2.081, p = 0.170 (See Figure 3A). The number of entries into the open arms did not differ between Oxtr −/− and Oxtr +/+ dams, F_1,13_ = 0.430, p = 0.522 (See Table 2). There was also no significant genotypic difference between the percent time in the inner versus outer portions of the arena for the open-field test, F_1,13_ = 2.418, p = 0.144 (See Figure 3B). There was a trend in the number of entries into the inner arena, with Oxtr −/− having fewer entries compared to Oxtr +/+ dams, F_1,3_ = 4.234, p = 0.060 (See Table 2). However, there was no significant difference in the total distance travelled between Oxtr −/− and Oxtr +/+ dams, F_1,13_ = 1.721, p = 0.212 (Table 2). There was also no genotypic difference in the percent swim/float for the forced swim test, F_1,17_ = 2.209, p = 0.156 (See Figure 3C).

**Table 2:** Normal anxiety-like and depression-like behavior in Oxtr −/− mice.

	Oxtr +/+ (mean ± SEM) (n = 8)	Oxtr −/− (mean ± SEM) (n = 9)
**Open field**		
Time in inner arena (%)	27.3±7.0	15.9±3.1
Time in outer arena (%)	72.7±7.0	84.1±3.1
Number of entries inner arena	135.7±29.4	73.6±11.9
Total distance traveled (cm)	5667.6±387.3	5060.3±556.6
**Elevated-plus maze**		
Time in closed arms (%)	80.5±4.9	64.5±9.3
Time in open arms (%)	19.5±4.9	35.5±9.3
**Forced Swim**		
Swim (%)	34.1±2.7	48.3±7.8

## Discussion

In the current study we used Oxtr −/− female mice to assess the importance of Oxt signaling in maternal behavior, anxiety-like, and depression-like behaviors in the postpartum period. Oxtr −/− females have increased pup abandonment, but those who initiated maternal behavior did not differ from wildtype thelectomized controls. These findings are consistent with past research that indicates that Oxt is important for the initiation, but not the maintenance of maternal behavior [43], [59]–[63]. For Oxtr −/− mice that did not abandon their pups, there were no genotypic differences in the following maternal behaviors measured: time on the nest, nest building, sniffing pups, grooming pups, or pup retrievals. Our data are consistent with pharmacological studies that have shown that Oxt can facilitate the initial onset of maternal behavior. Specifically, in virgin rats, icv injections of Oxt increase nurturing behavior directed towards pups [40], [43]. In postpartum rats, icv administration of Oxt antagonists or lesioning the PVN delays the onset of maternal behavior [41], [59], [60], [64], [65]. However, when Oxt antagonists are administered several days after the initiation of maternal care no deficits in maternal behavior are observed [64]. In Experiment 1, we found no deficits in Oxtr −/− dams' maternal care compared to Oxtr +/+ dams. However, 67% of offspring from Oxtr −/− dams were cannibalized or found dead by PND1, which suggests that once the maternal program is initiated that maternal behavior is normal.

Past research using Oxtr −/− and Oxtr FB/FB mice also support the hypothesis that Oxt is important to the initiation of maternal behavior. Previous studies have reported deficits in maternal behavior, as measured by longer latencies to retrieve pups, in Oxt −/− and Oxtr −/− mice [46], [49]. However, the aforementioned work was completed either using virgin Oxt −/− females fostering pups, or Oxtr −/− dams with pups fostered immediately following parturition [46], [49]. While work in the Nishimori line of Oxtr −/− mice found that PND0 Oxtr −/− mice have increased latencies to retrieve pups, we did not observe these differences in our line of Oxtr −/− mice on PND1-3 [49]. We did, however, observe that many of our Oxtr −/− dams abandoned their pups, approximately 67%. This finding is consistent with the pup abandonment phenotype observed in Oxtr FB/FB mice [50]. Overall, our data suggest that a functional Oxt system around the time of parturition lowers the threshold for the initiation of maternal behavior.

Given the design of Experiment 1, there are some potential limitations that should be considered. Based on observations that Oxtr −/− pups have reduced ultrasonic vocalizations compared to Oxtr +/+ pups [49], we had to account for the possibility that the pups themselves could contribute to a pup abandonment phenotype. In an attempt to control for this, we mated our Oxtr +/+ and Oxtr −/− females with male C57BL/6 mice or Oxtr Flox/Flox mice. Thus, none of the pups in our experiment had the −/− genotype. There is also the possibility that the wet nurses could contribute to pup mortality. However, since wet nurses were used for both Oxtr +/+ and Oxtr −/− mice, and there were large genotypic differences in the percentage of pup abandonment, it seems unlikely that the wet nurses could be solely responsible for the observed genotypic differences. Our data also suggest that Oxtr −/− dams were able to prevent wet nurses from providing maternal care by either facilitating abandonment or cannibalizing the pups. Lastly, there is the possibility that sham-thelectomized Oxtr −/− dams received different sensory input from pups attempting to nurse than thelectomized Oxtr +/+ controls, which in turn could have affected their maternal care. While this possibility cannot be ruled out, the rat literature suggests that thelectomy has no effect on non-nursing measures of maternal care or neuronal activation [52], [66], [67]. Specifically, while thelectomized rats display lower nursing durations than non-thelectomized controls [52], [66], [67], there are no observable differences in other components of maternal behavior, such as pup retrievals, licking and grooming behaviors, and time spent crouching over pups in thelectomized versus sham-thelectomized rats [52], [66], [67]. There also appear to be no thelectomy-dependent differences in c-fos activation in brain regions known to regulate maternal behaviors in rats [52], [53], [68]. However, while mice are not rats, the anatomy of the nipple, its innervation, and its neuroendocrine control are highly conserved in mammals (for review see [69]). So, while we cannot dismiss the possibility that there is a species difference in the effects of thelectomy on behavior, the literature suggests that differences in sensory input are not responsible for the lack of genotypic difference observed in this study.

Our finding of 67% of Oxtr −/− females showing pup abandonment is higher than the percentages reported in Oxtr FB/FB mice (only 40%–60% pup abandonment in their first litters) [50]. Perhaps the disruption of the Oxtr throughout the brain, rather than just in the forebrain, is functionally significant; this possibility will need to be explored further. It is unfortunate that we were not able to assess maternal aggression, but this is likely the result of our line of Oxtr mice being on a C57BL/6J background, which have been previously shown to have decreases in maternal aggression compared to other strains [70]–[72].

In Experiment 2, we found that Oxtr −/− dams exhibit no differences in postpartum anxiety-like or depression-like behaviors compared to Oxtr +/+ females. While there was a nearly statistically significant difference in the open field test, with Oxtr +/+ dams entering the inner arena more frequently than Oxtr −/− dams, we think that this is unlikely to be due to genotypic differences in anxiety-like behavior since no other behavioral measures indicated an anxiety-like phenotype. Also, the increased crossings do not appear to be the result of overall increased locomotion in the Oxtr +/+ females, as there was no genotypic difference in the total distance travelled. The lack of genotypic differences in the measures of anxiety was surprising given previous research indicating the anxiolytic effects of Oxt in the postpartum period [3], [28], [73], [74]. Following parturition, 2 of the 8 Oxtr +/+ and 7 of the 9 Oxtr −/− dams abandoned their pups by PND1; therefore the lack of contact with pups prior to the elevated plus and open field tests may have contributed to the absence of a genotypic difference in anxiety-like behavior. Statistical analysis could not be performed to determine if contact with pups prior to testing affected anxiety-like and depression-like behaviors, as only 2 Oxtr −/− dams did not abandon their pups. Further studies using only Oxtr +/+ and Oxtr −/− dams that were caring for pups may show different behavioral results. The lack of anxiolytic effects probably cannot be attributed to the absence of milk ejection since the absence of suckling by rat pups due to nipple removal does not affect a dam's anxiety; only relatively recent contact with pups prior to testing is thought to be necessary to observe reduced anxiety in the postpartum period [75].

Postpartum mood and anxiety disorders in humans are associated with impaired maternal care and a lack of attachment for the child [76], [77]. In humans, Oxt is known to promote bonding, and feelings of love and trust [78], [79]. In sheep, Oxt has been implicated in the formation of maternal bonds via olfactory recognition, allowing mothers to reject lambs that are not their own [80]. Higher plasma Oxt levels have been correlated with increased maternal bonding [38], and more recently a correlation has been shown between low plasma Oxt concentrations during pregnancy and the development of postpartum depression [39]. A role for Oxt in postpartum mood is supported by some recent work in Oxtr FB/FB dams where there is evidence of increased depression-like behavior in the postpartum period, with increased immobility times in the forced swim test and decreased sucrose ingestion relative to controls (A.H. Macbeth, personal communication). We, however, found no genotypic difference in depression-like behavior between Oxtr +/+ and Oxtr −/− dams. Due to the same issues raised previously, this may be a result of a lack of recent contact with the pups in many of our dams at the time of the forced swim test. In general, the relationship between recent pup interaction and depression-like behaviors in the postpartum period is not well studied, but lower depressed mood scores have been reported in human mothers who have had recent contact with their infants [81]. Therefore, future studies are still needed to address the role of Oxt in postpartum mood disorders, such as postpartum depression.

In summary, we have demonstrated that an absence of Oxt signaling results in increased pup abandonment in Oxtr −/− dams. However, in Oxtr −/− dams that did not abandon their pups, maternal behavior is normal. Also, in Oxtr −/− dams that have undergone parturition, there appear to be no profound effects on measures of anxiety-like or depressive-like behavior. Our research is consistent with the hypothesis that Oxt lowers the threshold for the initiation of maternal behavior, but that once the program is initiated Oxt is not critical to its maintenance.
